# Vitreoretinal Interface Characteristics in Eyes with Idiopathic Macular Holes: Qualitative and Quantitative Analysis

**DOI:** 10.4274/tjo.23327

**Published:** 2018-04-25

**Authors:** Arzu Seyhan Karatepe, Jale Menteş, E. Tansu Erakgün, Filiz Afrashi, Serhad Nalçacı, Cezmi Akkın, Yeşim Ateş

**Affiliations:** 1 Okan University Faculty of Medicine Hospital, Department of Ophthalmology, İstanbul, Turkey; 2 Ege University Faculty of Medicine, Department of Ophthalmology, İzmir, Turkey; 3 Kaşkaloğlu Eye Hospital, Ophthalmology Clinic, İzmir, Turkey; 4 Private Doctor, İzmir, Turkey

**Keywords:** Idiopathic macular holes, vitreoretinal interface, optical coherence tomography

## Abstract

**Objectives:**

To determine the qualitative and quantitative vitreoretinal interface characteristics with spectral domain optical coherence tomography (SD-OCT) in eyes with macular hole (MH) and investigate their relation with best corrected visual acuity (BCVA) and MH duration.

**Materials and Methods:**

Sixty-one eyes of 46 consecutive patients diagnosed with idiopathic MH were included in the study. The mean age of the patients was 66.7±7.5 (51-79) years. Complete ophthalmologic examination and SD-OCT examination were performed in all eyes and MH stages were determined according to SD-OCT findings. Qualitative characteristics of the vitreoretinal interface were investigated, including vitreomacular traction, vitreopapillary traction, maculopapillary traction, vitreoschisis, intraretinal cyst, presence of epiretinal membrane, and the integrity of the photoreceptor inner segment-outer segment junction (IS/OS) and external limiting membrane (ELM). In addition, MH diameter, MH base diameter (MHBD), ELM defect diameter, IS/OS defect diameter, and MH height were quantitatively measured and the MH index was calculated.

**Results:**

Out of 61 eyes, 9.8% were classified as stage 1a, 19.7% as stage 1b, 18% as stage 2, 23% as stage 3, and 29.5% as stage 4. Mean BCVA was 0.28±0.24 (1 mps-1.0) Snellen and MH duration was 10.08±18.6 (1-108) months. The most common interface characteristics associated with MH were determined as intraretinal cyst (91.8%), IS/OS defect (78.7%) and ELM defect (63.9%). Duration and stage of MH were inversely proportional to BCVA but directly proportional to the presence and diameter of IS/OS and ELM defects. BCVA was significantly lower in eyes with IS/OS and ELM defects (p<0.0001; p<0.0001 Mann-Whitney U test).

**Conclusion:**

We determined that the most important factors affecting BCVA in cases with idiopathic MH were MH stage, MH duration, MHBD, and the presence and diameter of IS/OS and ELM defects, which suggests that these parameters should be considered while making decisions about prognosis and treatment.

## Introduction

Macular hole (MH) is defined as full-thickness tissue loss in the central macula, including the internal limiting membrane (ILM) and photoreceptor layer. Though 80% of cases are idiopathic, MH is known to be one of the anomalies that arises during the development of posterior vitreous detachment (PVD).^1,2,3^ Mechanisms implicated in the pathogenesis of MH include abnormal interactions/adhesions between the posterior hyaloid membrane (i.e., the posterior cortical vitreous) and the macular surface (especially in the central fovea), as well as the forces exerted by anterior-posterior and tangential vitreous traction on the fovea.^4,5^

Spectral domain optical coherence tomography (SD-OCT) imaging of the retina, optic nerve head, and vitreomacular interface characteristics is currently regarded as an examination method that substantially facilitates our understanding of the course and pathogenesis of the disease.^6,7^ In addition to the diagnosis and clinical staging of idiopathic MH, SD-OCT enables *in vivo* imaging of various anatomical parameters and changes occurring in both the vitreomacular interface and retina, thereby providing insight in terms of prognosis and treatment.^8,9^

In this prospective clinical study, we aimed to use SD-OCT to examine the qualitative and quantitative characteristics of the vitreoretinal interface and inner and outer retinal layers in eyes with idiopathic MH, and investigate the relationships between these characteristics and best corrected visual acuity (BCVA) and MH duration.

## Materials and Methods

Sixty-one eyes of 46 consecutive patients diagnosed with idiopathic MH in the Retina Unit of the Ege University Medical Faculty Ophthalmology Department between January 2010 and January 2012 were included in the study. This prospective study was approved by the Ege University Ethics Committee (ETC-10-5.1/24) and written informed consent was obtained from all patients. Eyes with concomitant retinal conditions such as age-related macular degeneration, diabetic retinopathy, retinal detachment, or vascular diseases and eyes with uveitis, intraocular inflammation, or history of trauma or vitreoretinal surgery were excluded. 

MH duration was accepted as time from the start of reduced vision (as reported by the patient) to the date of presentation. All patients underwent full ophthalmologic examination and vitreous/retinal examinations with non-contact and contact lenses. In addition, SD-OCT (Topcon 3D OCT-2000; Topcon Europe Medical BV, Rotterdam) images of a 6x6 mm macular area and 3x8 mm area including the macula and optic nerve head were acquired in vitreous mode. Presence and extent of PVD were evaluated by clinical examination, SD-OCT, and B-scan ultrasonography (USG).

MH stage classification was based on SD-OCT findings^8^ as follows: stage 1a, presence of a cyst in the inner retinal layers at the central fovea due to vitreofoveal traction; stage 1b, extension of the cyst into the outer retinal layers, leading to a break in the outer retinal layers; stage 2, tractional break in the roof of the cyst, which forms a flap over a full-thickness hole; stage 3, full-thickness MH with free operculum and posterior vitreous attached to the optic disc; and stage 4 is characterized by a stage 3 MH with complete PVD.

**Figure 1:**
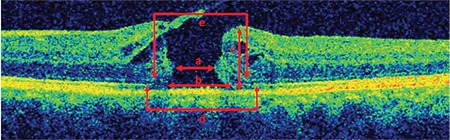
a) Macular hole (MH) diameter, b) MH base diameter, c) MH height, d) inner and outer segment defect diameter, e) external limiting membrane defect diameter

Qualitative characteristics of the vitreoretinal interface assessed in this study included vitreomacular traction (VMT), vitreopapillary traction (VPT), maculopapillary traction (MPT), vitreoschisis, intraretinal cyst, presence of epiretinal membrane (ERM), integrity of the photoreceptor inner segment/outer segment (IS/OS) junction, and external limiting membrane (ELM). In addition, MH diameter (MHD), MH base diameter (MHBD), ELM defect diameter, IS/OS defect diameter, and MH height (MHH) were quantitatively measured, and the MH index (MHI) was also calculated using the formula MHI=MHH/MHBD (Figure 1).

### Statistical Analysis

Correlations between BCVA and MH duration, MH stage, and quantitative and qualitative parameters of the vitreoretinal interface and inner/outer retinal layers were evaluated statistically. Kruskal-Wallis, Mann-Whitney U, chi-square, and Spearman tests were used for statistical analyses, and p value <0.05 was considered statistically significant.

## Results

The patient group comprised 30 females (65.2%) and 16 males (34.8%) with a mean age of 66.7±7.5 (51-79) years. Seventeen patients (37%) had bilateral MH. Thirteen (21.3%) of the eyes were pseudophakic and 48 (78.7%) were phakic.

MH stage was 1a in 6 eyes (9.8%), stage 1b in 12 (19.7%), stage 2 in 11 (18%), stage 3 in 14 (23%), and stage 4 in 18 (29.5%) of the eyes. Mean BCVA was 0.28±0.24 (counting fingers at 1 m - 1.0) Snellen and the mean MH duration was 10.08±18.6 (1-108) months. BCVA and MH duration according to MH stage are shown in Table 1. There were significant negative correlations between BCVA and MH stage and duration (p<0.0001, p<0.0001; Kruskal-Wallis test).

The distribution of vitreomacular interface characteristics such as VMT, VPT, MPT, vitreoschisis, intraretinal cyst, presence of ERM and IS/OS defects, and ELM defects according to MH stage is shown in Table 2. The most common interface characteristics coexisting with MH were intraretinal cyst (91.8%), IS/OS defect (78.7%), and ELM defect (63.9%). BCVA was not significantly correlated with VMT, VPT, MPT, vitreoschisis, intraretinal cyst, or presence of ERM (p=0.643; p=0.896; p=0.643; p=0.9; p=0.291; p=0.628, respectively; Mann-Whitney U test). However, eyes with IS/OS defect or ELM defect had significantly lower BCVA (p<0.0001; p<0.0001 Mann-Whitney U test). The prevalence of IS/OS defect and ELM defect was 0% and 16.7% in stage 1a eyes; 50% and 50% in stage 1b; and 100% and 100% in stage 4 eyes, respectively (Table 3). The presence and frequency of outer retinal layer defects increased significantly with higher MH stage (p<0.0001, p<0.0001; chi-square test). However, there were no significant correlations between MH stage and vitreoschisis, intraretinal cyst, or ERM presence (p=0.893, p=0.097, p=0.222; chi-square test) (Table 3).

Quantitative measurements of MHD, MHBD, MHH, MHI, ELM, IS/OS defect diameters, their distributions based on MH stage, and correlations with BCVA are shown in Table 4. There was no significant relationship between MHD and MH stage (p=0.192); however, MH stage was positively correlated with MHBD and MHH and negatively correlated with MHI (p=0.001, p <0.0001, p=0.011, Kruskal-Wallis test). Although BCVA and MHD were not statistically associated (p=0.974), BCVA levels were negatively correlated MHBD and MHH and positively correlated with MHI (p=0.002, r=-0.701; p=0.013, r=-0.589; p=0.018; r=0.566; Spearman test). 

Mean IS/OS defect and ELM defect diameters were 1327.7±822.5 µ and 1180.4±745.4 µ, respectively, and MH stage was positively correlated with defect diameter (p<0.0001, p=0.001; Kruskal-Wallis test). There were negative correlations between BCVA and IS/OS and ELM defect diameters (p=0.011, p=0.001, Spearman test).

The SD-OCT and B-scan USG data in our study were not correlated in terms of presence of complete PVD (Kappa=-0.19); a total of 22 eyes (36%) were diagnosed with PVD according to USG and 26 (42.6%) with SD-OCT (Table 5).

## Discussion

In the present study, we utilized SD-OCT to investigate the qualitative and quantitative characteristics of both the vitreoretinal interface and inner/outer retinal layers in a total of 61 eyes from 46 patients with idiopathic MH, and we evaluated correlations between morphological findings and functional status. 

Our results revealed statistically significant negative correlations between BCVA and MH stage and duration. Similarly, in a large-scale study conducted by Ho et al.^5^, visual acuity decreased as the MH stage and duration increased.

Furthermore, we identified intraretinal cyst, IS/OS defect, and ELM defect as the most common concomitant interface characteristics in our MH patients. Huang et al.^8^ reported intraretinal cysts in 93% of their idiopathic MH cases. Scholda et al.^9^ examined the presence of IS/OS defect and intraretinal cyst in eyes with MH using ultrahigh resolution OCT. They reported that eyes with more intraretinal cysts had larger IS/OS defects and attributed this to the intraretinal cysts blocking reflectance in that region. Although we did not observe any significant relationships between BCVA and VMT, VPT, MPT, vitreoschisis, intraretinal cyst, or ERM in our MH cases, BCVA was significantly lower in patients with IS/OS and ELM defects. In addition, we detected IS/OS and ELM defects even in eyes with stage 1a MH; the frequency of these defects was 38.8% and 33.3% respectively in stage 1 eyes and 100% in stage 4 eyes. We observed a significant positive correlation between MH stage and the presence and frequency of these defects.

Although MHD was not significantly associated with BCVA or MH stage, we noted that MHBD and MHH were significantly greater and MHI lower at higher MH stages. Similarly, higher MHBD and MHH and lower MHI correlated with lower BCVA. In a study including stage 2, 3, and 4 MH cases, Wang et al.^10^ reported a positive association between MHD and stage. The diameter referred to in that study was MHBD and the data were consistent with those of our study. Chew et al.^11^ also reported larger MHBD and lower BCVA levels with longer MH duration. They also noted a quantitative increase in IS/OS and ELM defect diameters and decreased BCVA levels with higher MH stage. Consistent with our study, Chang et al.^12^ reported MHD and IS/OS defect diameter as the main parameters affecting BCVA levels in 24 eyes with MH and 17 eyes with closed MH, with larger diameters associated with lower BCVA levels.

We observed that B-scan USG and SD-OCT findings were not strongly correlated for diagnosis of complete PVD in patients with MH. Kicova et al.^13^ used preoperative slit-lamp biomicroscopy, B-scan USG, and OCT to evaluate the presence of PVD in 30 eyes scheduled for vitrectomy due to MH or macular pucker. They reported that slit-lamp biomicroscopy and USG provided more accurate diagnostic results based on intraoperative findings.

## Conclusion

In summary, we determined that in idiopathic MH, key factors in visual acuity are the base diameter and height of the MH and the presence and diameter of IS/OS and ELM defects. These parameters were positively associated with MH stage and duration, and we recommend that these parameters be taken into consideration when determining prognosis and making surgical decisions.

## Figures and Tables

**Table 1:**
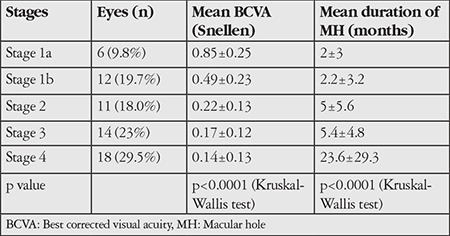
Best corrected visual acuity and macular hole duration of the eyes based on macular hole stage

**Table 2:**
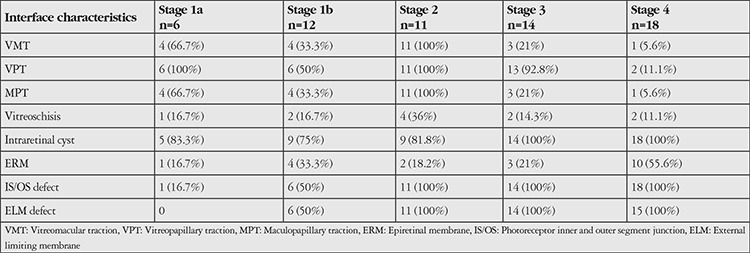
Qualitative vitreoretinal surface characteristics according to macular hole stage

**Table 3:**
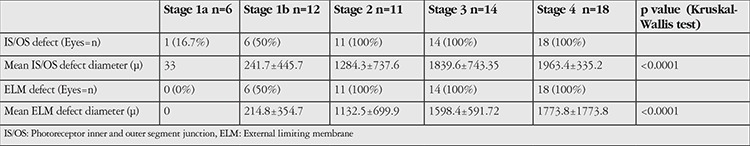
Inner and outer segment and external limiting membrane defect presence and size according to macular hole stages

**Table 4:**
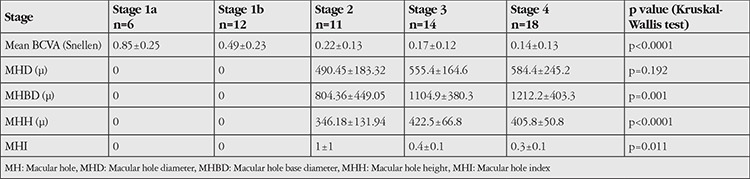
Quantitative macular hole characteristics and best corrected visual acuity according to macular hole stage

**Table 5:**
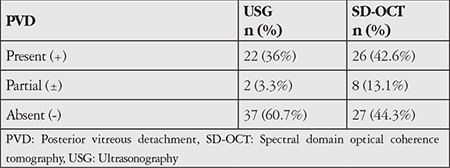
Presence of posterior vitreous detachment according to spectral domain optical coherence tomography and ultrasonography data
